# Effect of Specific mode electroacupuncture stimulation combined with NGF during the ischaemic stroke: Study protocol for a randomized controlled trial

**DOI:** 10.1016/j.clinsp.2024.100451

**Published:** 2024-07-20

**Authors:** Mengyuan Dai, Yibin Zhao, Zhaoxing Jia, Shiting Xu, Nuo Xu, Xuewen Wu, Jianxun Liu, Lixiu Wu, Kunqiang Yu, Xianming Lin

**Affiliations:** a The Third Clinical Medical College, Zhejiang Chinese Medical University, Hangzhou, Zhejiang Province, China; b Department of Rehabilitation, The Second People's Hospital of Lishui, LiShui, ZheJiang Province, China; c Zhejiang Provincial Hospital of Chinese Medicine, The First Affiliated Hospital of Zhejiang Chinese Medical University, Hangzhou, Zhejiang Province, China; d Department of Rehabilitation, Ningbo No.2 Hospital, Ningbo, Zhejiang Province, China; e The Third Affiliated Hospital of Zhejiang Chinese Medical University, Xihu District, Zhejiang Province, China; f Department of Rehabilitation, Zhejiang Rehabilitation Medical Center, Hangzhou, Zhejiang Province, China

**Keywords:** Electroacupuncture, NGF, Ischemic stroke, Study protocol

## Abstract

**Background:**

The emergence of the Nerve Growth Factor (NGF) has promoted the development of neuroprotective therapy; however, it has little effect on cerebral ischemia because of its poor Blood-Brain Barrier (BBB) permeability. Specific Mode Electroacupuncture Stimulation (SMES) can open BBB safely and effectively; however, it has shown inconclusive clinical effects and indirect clinical evidence in the recovery phase. Hence, the authors conducted a multicentre, randomized, placebo-controlled, assessor-blinded clinical trial to assess the effectiveness and safety of SMES combined with NGF treatment used during ischaemic stroke recovery.

**Methods:**

A total of 288 stroke patients from three hospitals will be recruited and randomly allocated to four groups: acupuncture + placebo, acupuncture + NGF, SMES + placebo, and SMES + NGF, in a 1:1:1:1 ratio. Assessment data will be collected at baseline, 2-weeks, and 4-weeks during the treatment period, as well as at the 4-week and 8-week follow-up after treatment completion. The primary outcome measure will be the basic cure rate. The secondary outcome measures include the simplified Modified Barthel Index, Timed Up and Go Test, Fugl-Meyer Assessment of Motor Function Score, Tinetti Performance Oriented Mobility Assessment, Montreal Cognitive Assessment, and Loewenstein Occupational Therapy Cognitive Assessment. Moreover, resting-state functional magnetic resonance imaging and Functional near-infrared spectroscopy can detect changes in cerebral blood flow and brain function and investigate the relationship between the clinical efficacy and mechanism of the prescribed interventions.

**Conclusion:**

This study will provide clinical evidence for the efficacy and safety of SMES combined with NGF in the treatment of stroke patients.

## Highlights

• This protocol describes a multicentre, randomized, placebo-controlled trial that aims to assess the efficacy and safety of specific stimulation mode SMES combined with NGF in reducing the recovery period of ischaemic stroke and provide evidence-based medical data.
• This research will help provide the latest and high-quality evidence for clinicians and policymakers seeking innovative and effective ways to treat patients with ischaemic stroke.

## Introduction

A trend towards increasing stroke incidence or mortality rates, in people was recently observed in China.^1^ Ischaemic stroke is a common clinical disease that mainly manifests as limb movement and sensory disorders accompanied by decreased learning and memory ability.^2^ A large number of clinical data show that the ischaemic stroke during convalescence, which ranges from 2-weeks to 6-months after the onset of stroke, is the best time for functional recovery of patients with cerebral infarction.^3^ Therefore, improving the clinical efficacy of stroke treatment during the recovery period after stroke is a widely researched topic of interest in neuroscience.

Nerve Growth Factor (NGF) was the first neurotrophic drug to be discovered; it provides nerve repair and protective effects as well as plays an active role in nourishing nerves, promoting neuronal survival, and inhibiting cell apoptosis.^4^ A recent case report demonstrated that intranasal NGF administration improved cerebral function in a child with severe traumatic brain injury.^5^ Nevertheless, the effect of NGF on patients with ischaemic stroke during convalescence is not satisfactory as the molecular weight of NGF is approximately 13.4 kDa, which makes it difficult for it to cross the Blood-Brain Barrier (BBB) under normal conditions and affects its ability to reach the effective drug concentration to exert therapeutic effect.^6^ Therefore, there is an urgent need to find an effective method through which neuroprotective agents, such as NGF, can safely penetrate the BBB.

Acupuncture is a method that has been employed to treat stroke in China since ancient times and has been widely accepted worldwide.^7^ Specific Mode Electroacupuncture Stimulation (SMES) involves the application of electrical stimulation in which an electric current passes between acupuncture needles.^8^ Previous studies have shown that SMES can be used as a means of opening the BBB. Moreover, our previous study showed that at a frequency of 2/100 Hz, 3 mA SMES (a homemade relay cycled power to the electrode with 6s on-and off-cycle) applied for 40 min at the meridians GV20 (Baihui) and GV26 (Shuigou) can increase the permeability of BBB to NGF.^9^ Our repeated experiments on SD rats revealed that these SMES stimulation parameters were stable and safe for the opening of the BBB; therefore, our research team named it specific stimulation mode SMES.

This study aimed to evaluate the effectiveness and safety profile of specific stimulation mode SMES combined with NGF used in the treatment of ischaemic stroke during the recovery period. The main hypothesis of this trial is that compared to the control groups, the SMES + NGF group will provide remarkably improved motor and cognitive function. Moreover, these improvements will be associated with changes in cerebral blood flow and brain function. Further, this study innovatively combines SMES and NGF to treat patients with ischaemic stroke during convalescence and, thus, will provide a new treatment method with sufficient clinical evidence.

## Methods and analysis

### Study design

**Fig. 1:**
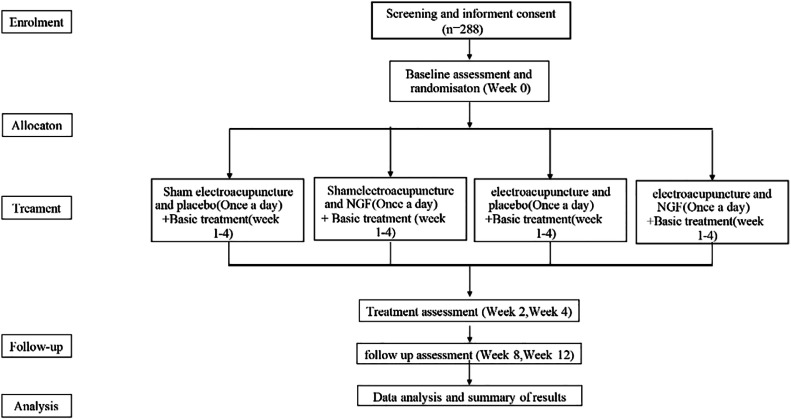
Study Flow: Steps that all patients should go through from enrolment to analysis.

This study will be a multicentre, parallel, outcome-assessor-blinded, placebo-controlled, randomized trial consisting of a 4-week treatment phase, followed by a 12-week follow-up phase. Figure 1 displays the trial process, and Table 1 provides details of the trial schedule.

**Table 1:** Schedule of enrolment, interventions and assessments.

Stage	Baseline	Treatment phase				Follow-up phase	
Number of visits	1	2	3	4	5	6	7
Time point	Week 0	Week 1±1 day	Week 2±1 day	Week 3±1 day	Week 4±1 day	Week 8±3 days	Week 12±3 days
Eligibility screening	X						
Informed Consent Form	X						
Demographic characteristics	X						
Medical history	X						
Definitive diagnosis	X						
Inclusion criteria	X						
Exclusion criteria	X						
Evaluation of treatment expectation	X						
Therapeutic effect record form		X	X	X	X		
Scale assessment							
Modified Rankin Scale, mRS	X		X		X	X	X
Modified Barthel Index (MBI)	X		X		X		
Simplified Fugl-Meyer Assessment of motor function score (FMA)	X		X		X		
Timed up and go test	X		X		X		
Tinetti Performance Oriented Mobility Assessment (POMA)	X		X		X		
Montreal Cognitive Assessment (MoCA)	X		X		X	X	X
Informant Questionnaire on Cognitive Decline in the Elderly (IQCODE)	X						
Mini-mental State Examination, MMSE	X						
Loewenstein Occupational Therapy Cognitive Assessment (LOTCA)	X		X		X		
Acupuncture safety evaluation		X	X	X	X		
NGF treatment safety evaluation		X	X	X	X		
Adherence assessment					X		
Combination of medications	X						
Adverse Events		X					
Data Processing	X						
Research Completion	X						
Researcher Review	X						

### Setting, recruitment, and participants

This trial will be conducted at the Third Affiliated Hospital of Zhejiang Chinese Medical University, the Ningbo Hwamei Hospital, and the Lishui Second People's Hospital. The recruitment criteria will include:

#### Inclusion criteria

The inclusion criteria include (1) Patients with first-ever ischaemic stroke confirmed by Computed Tomography (CT) and/or Magnetic Resonance Imaging (MRI); (2) 14-days to 6 months after the onset of stroke;(3) Patients between the ages of 30 and 80; (4) Modified Rankin Score (mRS), 3‒4; and Mini-Mental State Examination (MMSE) < 27; (5) Patients who are able to accept and comply with EA treatment and have good compliance; (6) A willingness to participate in the project.

#### Exclusion criteria

The exclusion criteria are: (1) Transient ischaemic attack, cerebral haemorrhage, and other cerebrovascular diseases; (2) Unstable medical condition (e.g., patients in the intensive care unit, myocardial infarction, severe pulmonary disease); (3) Brain tumours, cerebral infarction caused by repeated recurrence of stroke and brain surgery or trauma; (4) Visual or auditory impairment, aphasia, agnosia, severe hemiplegia, or affected limb function before stroke; (5) Deficiencies in cognitive function prior to stroke, such as alcoholism, drug abuse and congenital dementia, Informant Questionnaire on Cognitive Decline in the Elderly (IQCODE) ≥ 3.19; (6) Women who are lactating, pregnant or intending to get pregnant; (7) Skin infection at acupuncture site, needle sickness, or needle phobia; (8) Implantable cardioverter defibrillator carriers, conductive metallic foreign bodies in the body, or a pacemaker; (9) Allergic after using NGF; (10) Participated in clinical trials three months ago or currently in other clinical trials.

### Randomization and allocation concealment

Patients who qualify for inclusion will be recruited from March 2022 to December 2025. The recruited participants will be randomly divided into four groups by a randomization system (eBalance, Randomization and Trial Supply Management, Taimei Technology, Shanghai, China) and allocated to four groups, namely: acupuncture + placebo, acupuncture + NGF, SMES + placebo, and SMES + NGF groups.

### Blinding

In this trial, it will be difficult to blind the researchers because they will perform SMES or acupuncture procedures and inject NGF or placebo into patients. Throughout the trial, patients, independent statisticians, and independent outcome assessors will be unaware of the group allocation data.

### Interventions

#### Acupuncture intervention

**Fig. 2:**
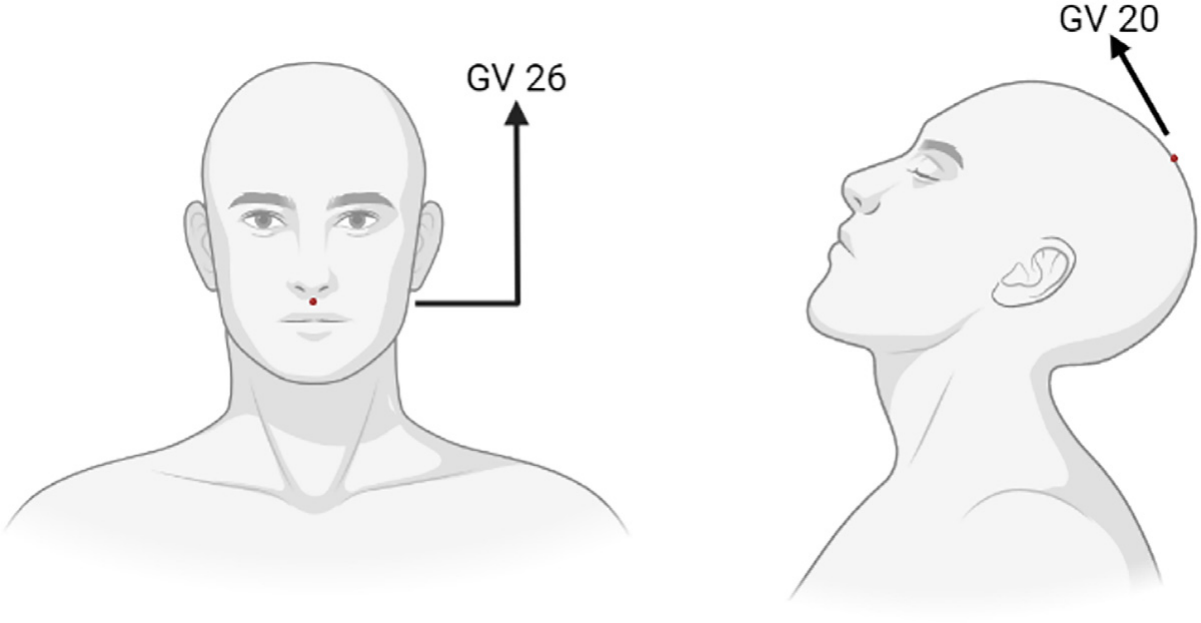
Location diagram of GV20 (Baihui) and GV26 (Shuigou).

The patient lay in a supine position, after disinfection of acupoint skin, two stainless steel needles (Hua Tuo brand, Suzhou Medical Supplies Company Ltd., Jiangsu, China) of sizes 0.25 × 40 mm and 0.25 × 25 mm, will be inserted into the GV20 (Baihui) and GV26 (Shuigou), respectively (Fig. 2). The acupoints will be stimulated manually until the patients feel distension, heaviness, or soreness.

#### SMES intervention

On the basis of the above acupuncture, add the electroacupuncture instrument (HANS-200, Nanjing Jinsheng, Ltd., China) to connect the acupuncture needle with an intensity of 3 mA and a frequency of 2/100 Hz for 40 min (a self-made relay cycled power to the electrode for 6s on and off).

#### NGF injection

A 20 µg NGF (n° S20060051, Jinlujie, Hiteck Biopharmaceutical Co., Ltd., Wuhan, China) will be dissolved in 2 mL sterile water for injection. Patients will receive intramuscular injection of gluteus maximus once a day, 28 days a course of treatment according to the instructions.

#### Study flow and treatment procedure

NGF or placebo will be injected into the gluteus maximus and followed by either SMES or acupuncture for 40 min.

#### rs-fMRI

A reasonable number of acupuncture + NGF and SMES + NGF (i.e., 20) will be selected. These patients will then undergo an rs-fMRI examination using a 1.5T MRI scanner (uMR586, Shanghai Lianying Medical Technology Co., Ltd., China). Patients will be required to remain still, close their eyes, remain awake and relaxed, and minimize their thoughts.

The resting-state rs-fMRI will sequence for obtaining Blood-Oxygen-Level Dependent (BOLD) and Three-Dimensional T1-Weighted Imaging (3D-T1WI). After data processing, the values of the Amplitude of Low-Frequency Fluctuations (ALFF), Regional Homogeneity (ReHo), and Functional Connectivity (FC) will be calculated to observe changes in brain function.

#### fNIRS

Each group will select a sample size of 10 patients with infarct lesions on the left side. The fNIRS examination will be conducted using the LIGHTNIRS device manufactured by Shimadzu in Kyoto, Japan.

Patients will perform three tasks sequentially: cognitive, upper limb movement, and lower limb movement tasks. The interval between tasks will be 2 min. The Cognitive task requires completing the Stroop task, an event-related design using E-prime 3.0 (Psychology Software Tools Inc., Pittsburgh, PA, USA).^10^ The upper-limb movement task consists of clench tasks (10 times) with a disabled hand. Lower limb movement tasks include bilateral hip joint exercises (10 times) using a mechanical pedal. Each task comprises 15s of movement execution and 15s of rest.

After data collection, fNIRS data will be pre-processed and analyzed using the Metlab 2020b (MathWorks Inc., Natick, Massachusetts, United States).^11^

### Outcome measures and evaluation

The primary outcome measure will be the basic cure rate (patients with mRs ≤2 are judged as clinically cured; basic cure rate = number of participants in a group with mRs ≤2/total participants in group × 100%).^12^ The mRs score was 0‒6 points. The higher the score, the more serious the neurological deficit. The secondary outcome indicators are FMA, MBI, TUGT, POMA, MoCA, LOTCA.

Baseline data will be collected by an independent outcome evaluator on the first visit (week 0), including participants' demographic data, medical history, comorbidities, major medications, and clinical evaluation, including ECG, routine blood/urine/stool tests, electrolytes, liver/renal function examinations, as well as mRs, FMA, MBI, TUGT, POMA, MoCA, and LOTCA. At the end of treatment (week 4), patients will undergo the above examinations again and subsequently enter the follow-up period.

### Follow-ups

All participants in the four groups will be contacted either through outpatient, home visit, telephone, or other forms of communication according to the different physical conditions of the patients by the same outcome assessor at 4- and 8-weeks after the trial. The outcome assessor will use the mRs and MoCA scales to assess patients' conditions during the follow-up phase.

### Safety assessment

All patients will be requested to report Adverse Events (AEs) that occur during the trial. AE details, including duration, severity, measures taken, and outcome, will be recorded in Case Report Forms (CRFs). The relationship between the intervention and AEs will be assessed according to the WHO Uppsala Monitoring Centre System for standardized case causality assessment.^13^ If patients develop a severe AE (SAE) related to SMES or NGF during the trial, they will immediately obtain medical help and be allowed to drop out of the trial. Severe Adverse Reactions (SARs) or SAEs are defined according to the International Conference on Harmonisation Tripartite guidelines and will be reported to the ethics committee of the Third Affiliated Hospital of Zhejiang Chinese Medical University and the research team.^14^

### Data collection, management, and monitoring

Patients' information, results from the scores' evaluation, laboratory examination, and other relevant data will be collected at baseline (week 0), post-treatment (week 4), and follow-up assessments (weeks 4 and 8 after the trial). AEs will be observed and recorded from the baseline to the final follow-up. The data will be entered into a pre-designed database by two independent investigators. The data entered will be scrutinized by another researcher to ensure accuracy.

### Sample size calculation

Based on our earlier randomized controlled pre-study, the authors use The basic cure rate as the baseline for the sample size calculation for the present work; the rate of acupuncture + placebo, acupuncture + NGF, SMES + placebo, and SMES + NGF groups were 35%, 40%, 50%, and 75%, respectively.to observe the difference in the basic cure rates between the 4 groups of cases were distributed in proportion of 1:1:1:1, setting the significance value as 5% (α = 0.05), and the test efficiency with 1-β = 0.8. Four groups of samples were calculated, with at least 55 samples in each group. Considering the existence of factors, such as shedding and loss to follow-up, the sample size was expanded by 20%. Finally, the sample was expanded to 288 cases. The sample size estimation formula used is as follows (here PT = 75%, PC = 50%): $n=\frac{{\left(z_{\alpha }+z_{\beta }\right)}^{2}[p_{c}\left(1-p_{c}\right)+p_{r}\left(1-p_{r}\right)}{{\left(p_{r}-p_{c}\right)}^{2}}$.

### Statistical analysis

Statistical analysis will be conducted by an independent statistician using SAS V. 9.3 (SAS Institute, Cary, North Carolina, USA) and the Statistical Package for the Social Sciences software V. 26.0 for Windows (IBM SPSS Statistics, IBM Corp., Somers, New York, USA) will be used for the data analyses. For all analyses, the statistical significance level will be set at 5% (α = 0.05).

## Discussion

The current treatment protocols for ischaemic stroke are focused on acute ischaemic stroke, including thrombolytic therapy, antiplatelet aggregation, and neuroprotective therapy, which benefit only a small population of patients with stroke because of the narrow therapeutic time window.^15^ Most patients with ischaemic stroke are in the recovery period with obvious limb dysfunction and cognitive impairment; The intensity of interventions that have been studied to date might be inadequate.^16^

Acupuncture is recommended as a treatment for stroke in traditional Chinese medicine, and its safety has been confirmed by a wide variety of literature.^9^^,^^17^^,^^18^ However, evidence of its efficacy in previous studies has been inconclusive regarding the treatment of patients with ischaemic stroke in the recovery period.^19^ NGF, the first neurotrophic drug to be discovered, plays an active role in regulating neuronal development, differentiation, plasticity, cell death, and survival. However, NGF is mainly used for the treatment of peripheral neuropathy and is rarely used during the recovery period of ischaemic stroke.^20^^,^^21^ This is because the barrier between the brain and blood, BBB, is an obstacle to the transportation of large molecules into the brain, including those used for treating diseases of the central nervous system such as stroke and Alzheimer's Disease (AD).^22^ An intact BBB under physiological conditions and a repaired BBB under pathological conditions prevent the entry of peripheral inflammatory substances and regulate the exchange of toxins and nutrients between the brain and blood.^23^

Based on the above reasons, several studies have found that SMES is similar to an intervention method for opening the BBB and inducing the transportation of drugs, such as NGF, into the brain. Furthermore, the authors found that the opening effect on the BBB was most obvious under SMES intervention at the GV20 and GV26 meridian points.^9^ As a result, the authors selected the optimal stimulus parameter of specific stimulation mode SMES, which can contribute to the development of neuroprotective therapy, especially for facilitating the use of NGF.^24^

Stroke rehabilitation training and evaluation play important roles in stroke treatment. Patients are usually provided individual rehabilitation training plans according to their condition; therefore, the authors will strictly follow the uniform criteria for rehabilitation evaluation. The mRs can be considered the strongest predictor of subsequent quality of life.^25^ The FMA reflects the level of motor dysfunction and is widely used in the evaluation of motor dysfunction after stroke.^26^^,^^27^ The MBI is a common method for assessing activities of daily living, and the TUGT mainly calculates the time required to stand up for a walk to assess motor function in patients.^28^ The POMA scale is a useful tool for evaluating physical function and fall risk in stroke survivors.^29^ Compared with MMSE, The MoCA and LOTCA are more comprehensive and, be used clinically for cognitive impairment detection.^30^^,^^31^ Moreover, rs-fMRI is a powerful method of examining the networks of neurons involved in motor and cognitive processing and the effects of disease status on brain function.^32^ fNIRS works by using optical methods to measure changes in cerebral blood flow and oxygen saturation.^33^ Therefore, evaluation of the scales combined with rs-fMRI and fNIRS can further explain the relationship between the improvement of clinical efficacy and changes in brain function.

There are a few limitations to this study. First, the sample size calculation was based on the data from our pilot trial, which may affect the accuracy of the sample size calculation results. Second, the basic cure rate is calculated rather than considering the measurement of mRs as the primary outcome measure because efficacy assessment for the whole population during the study is necessary, and there should be scientific evaluation criteria; however, this may produce false results.

Under strict quality control, this study will potentially confirm the clinical efficacy of specific stimulation mode SMES combined with NGF for the recovery period of ischaemic stroke. This study also aims to explore the correlation between clinical efficacy and changes in brain function, which is of great significance in providing ample evidence to consolidate the theoretical basis for the application of this combined therapy.

## Conclusion

This protocol describes a multicentre, randomized, placebo-controlled trial that aims to assess the efficacy and safety of specific stimulation mode SMES combined with NGF in reducing the recovery period of ischaemic stroke and provide evidence-based medical data. The results of this research will help provide the latest and high-quality evidence for clinicians and policymakers seeking innovative and effective ways to treat patients with ischaemic stroke.

## Trial registration

ClinicalTrials.gov Identifier NCT05231694.

## Trial status

Patient recruitment will begin in March 2022 and is expected to finish at the end of 2025.

## Patient consent

Obtained.

## Ethics approval

The ethics committees of the Third Affiliated Hospital of Zhejiang Chinese Medical University, Ningbo Hwamei Hospital, and Lishui Second People's Hospital approved the study.

## Fund

The trial is funded by the Special Project of Modernization of Traditional Chinese Medicine in Zhejiang Province in 2022 (n 2022ZX009) and the National Natural Science Foundation of China (n 82174502).

## Data sharing statement

The data used to support the findings of this study have been deposited in the Clinical Trials repository whose ID is NCT05231694. The URL is https://clinicaltrials.gov/ct2/show/NCT05231694.

## Authors’ contributions

Mengyuan Dai: Conceptualization, Writing − original draft, Data curation. Yibin Zhao: Conceptualization, Writing − original draft. Zhaoxing Jia: Conceptualization, Writing − original draft. Shiting Xu: Supervision. Nuo Xu: Data curation. Xuewen Wu: Resources, Supervision. Jianxun Liu: Resources, Supervision. Lixiu Wu: Resources, Supervision. Kunqiang Yu: Resources. Xianming Lin: Investigation, Methodology, Funding acquisition, Writing − review & editing.

## Declaration of competing interest

The authors declare no conflicts of interest.
